# Prevalence, serologic and genetic studies of high expressers of the blood group A antigen on platelets*

**DOI:** 10.1111/j.1365-3148.2010.01017.x

**Published:** 2010-10

**Authors:** B M Sant’Anna Gomes, A C Estalote, M Palatnik, G Pimenta, B de B Pereira, E M do Nascimento

**Affiliations:** 1 Blood Transfusion Service Research Laboratory, Hospital Universitário Clementino Fraga Filho (HUCFF), Universidade Federal do Rio de Janeiro (UFRJ)Rio de Janeiro (RJ), Brazil; 2 Postgraduate Program on Clinical Medicine (Hematology), Faculty of Medicine, Universidade Federal do Rio de Janeiro (UFRJ)Rio de Janeiro (RJ), Brazil; 3 Division of Hematology, HUCFF, Universidade Federal do Rio de Janeiro (UFRJ)Rio de Janeiro (RJ), Brazil; 4 Scientific Investigation Committee, HUCFF, Faculty of Medicine, Universidade Federal do Rio de Janeiro (UFRJ)Rio de Janeiro (RJ), Brazil; 5 Postgraduate Programs in Engineering (COPPE), Universidade Federal do Rio de Janeiro (UFRJ)Rio de Janeiro (RJ), Brazil

**Keywords:** Platelets, ABO blood group, *ABO* gene, Genetic Polymorphism, High Expresser Platelet Phenotype

## Abstract

**Objective/Aim:** The aim of this study is to describe the distribution of the platelet blood group A antigenicity in Euro-Brazilians (EUBs) and Afro-Brazilians (AFBs).

**Background:** A small but significant proportion of individuals express high levels of A or B antigen on their platelets corresponding to the erythrocyte ABO group. The mechanism of increased antigen expression has not been elucidated.

**Material/Methods:** A cohort of 241 blood group A donors was analysed by flow cytometry. Although mean fluorescence intensity (MFI) is a typical continuous variable, platelets were screened and divided into two categories: low expressers (LEs) and high expressers (HEs). A three-generation family was investigated looking for an inheritance mechanism.

**Results:** The prevalence of the HE platelet phenotype among group A_1_ donors was 2%. The mean of MFI on platelets of A_1_ subgroup of EUBs differs from that of AFBs (*P* = 0·0115), whereas the frequency of the HE phenotype was similar between them (*P* = 0·5251). A significant difference was found between sexes (*P* = 0·0039). Whereas the serum glycosyltransferase from HE family members converted significantly more H antigen on group O erythrocytes into A antigens compared with that in LE serum, their *ABO*, *FUT1* and *FUT2* genes were consensus. The theoretically favourable, transcriptionally four-repeat *ABO* enhancer was not observed.

**Conclusion:** The occurrence of HE in several members suggests familial aggregation. Indeed, in repeated measures, stability of the MFI values is suggesting an inherited condition. Factors outside the *ABO* locus might be responsible for the HE phenotype. Whether the real mechanism of inheritance is either of a polygenic or of a discrete Mendelian nature remains to be elucidated.

Human platelets express alloantigens that are platelet specific (e.g. Human Platelet Antigens (HPA)) and others that are shared with other blood cells and tissues (e.g. ABH, P, Le, I and Human Leucocyte Antigens (HLA) class I determinants) (Kunicki & George, 1989). Although it has been known for decades that human platelets express A and B antigens corresponding to the ABO blood group of the individual's erythrocytes, many blood banks worldwide transfuse platelets without regarding donor–recipient ABO compatibility. Although this practice might relieve inventory pressures, several reports have described platelet refractoriness mediated by anti-A and anti-B antibodies (Skogen *et al.*, 1988; Ogasawara *et al.*, 1993; Curtis *et al.*, 2000).

In Japan, approximately 7% of the population expresses significantly elevated levels of either A or B antigen on the platelet surface (Ogasawara *et al.*, 1993). It has been suggested that the expression of ABH antigens on platelets is genetically determined and that individuals can be classified as either low expressers (LEs) or high expressers (HEs) depending on the amount of A or B antigen detectable on their platelets. Curtis *et al.* (2000) showed that platelet A antigen levels in 7% of Caucasian blood group A_1_ donors were higher than in the general population mean + 2SD using flow cytometry. The corresponding percentage for B antigen in B donors was 4%. Furthermore, these investigators proposed that HE individuals could be divided into two categories of HEs of group A or B antigen, based on the flow cytometrically generated histogram pattern. The amount of these antigens on the platelets in the HE type I (HE-I) group overlapped with the mean of that in the general population but was, on average, higher. In the HE type II (HE-II) group, there was a clear separation between the amount of platelet A (or B) antigen on these donors' platelets and the mean of the general population as shown by the histogram.

A recent study indicated that individuals with the A_2_ red blood cell (RBC) phenotype may lack A antigen as well as its precursor substance H antigen on their platelets (Cooling *et al.*, 2005), although this finding has been questioned by others (Curtis & Aster, 2006). We have evaluated the amount of A antigen on the platelet surface of 241 Brazilian blood group A_1_, A_2_ and A_int_ donors. This is the largest cohort of A donors studied so far and also the first report about ethnic groups. We also present a study on a group A_1_ HE Afro-Brazilian (AFB) family with a complete analysis of the underlying *ABO* gene sequence including its 5′ enhancer region, suggested by some investigators (Gershan *et al.*, 2006) to play a role in *ABO* transcriptional platelet regulation. The *FUT1* and *FUT2* genes, which are involved in synthesising the ABO precursor substance (H antigen) on RBCs and in secretions, respectively, have also been investigated. The occurrence of HE phenotype in several members suggests familial aggregation of the character. The potential mechanism of inheritance of the platelet HE phenotype is also discussed.

## MATERIALS AND METHODS

### Specimens

On giving blood, 241 Brazilian group A blood donors agreed to participate in the study. The study was approved by the Ethical Scientific Committee (Comitê de Ética em Pesquisa, registration number: 001/003, Hospital Universitário Clementino Fraga Filho/ Faculdade de Medicina, Universidade Federal do Rio de Janeiro, Brazil), and all subjects received oral and written information concerning the study and gave their informed consent. The ethnicity of the donors, either Euro-Brazilian (EUB) or AFB, was assigned at the time of blood donation, as previously reported (Palatnik *et al.*, 2002). Furthermore, a possible bone marrow donor, who had shown the highest level of platelet A antigen, and his 13 living relatives were also analysed. Informed consent was obtained from each individual or from the parents on behalf of their children. All of the family members were AFBs born in Rio de Janeiro, Brazil.

Following Michelson *et al.* (2000), for platelet studies, a 4-mL venous blood sample was collected from each individual into 3·2% sodium citrate-coated polyethylene terephthalate tubes (Vacuette^®^, Greiner Bio-One, Americana, SP, Brazil). Saliva and additional blood samples (8 mL in ethylenediaminetetraacetic acid and 10 mL in tubes without additives) from family members and some blood donors for platelet ABO antigen reproducibility tests, glycosyltransferase (GTA) assays and genetic testing were also obtained as described in subsequent sections.

### Erythrocyte phenotyping

The donors' blood groups were initially determined by slide test with monoclonal anti-A and anti-B reagents (DiaMed, Lagoa Santa, MG, Brazil) and repeated using the microplate system (DiaMed-MP Test^®^, DiaMed, Cressier sur Morat, Switzerland). To differentiate between the A subgroups, anti-A_1_ (*Dolichos biflorus*, Biotest, Itapecerica da Serra, SP, Brazil, and mouse hybridoma, Biotest, Dreieich, Germany) and anti-H (our own crude extracts of *Ulex europaeus* seeds and a commercial lectin from Biotest, Brazil) testing were carried out according to the manufacturers' instructions. The definitions of the A subgroups followed published recommendations (Palatnik, 1984; see also Daniels, 2002).

### Platelet preparation

To avoid *ex vivo* platelet activation, samples were processed within 30 min of blood drawing for all assays (Mody *et al.*, 1999; Michelson, 2006). Each blood sample was centrifuged at 22 °C for 5 min at 500 ×*g* in a Jouan Model BR 4i centrifuge (Societe Jouan, St Herblain, France). To minimise the formation of platelet aggregates, one part of the plasma supernatant (platelet-rich plasma, PRP) was diluted in two parts of 4-(2-hydroxyethyl)-1-piperazineethanesulfonic acid (HEPES)-buffered saline [0·14 M NaCl, 5 mm KCl, 1 mm MgSO_4_ and 10 mm HEPES (sodium salt), pH 7·4]. Samples were stabilised by fixation in paraformaldehyde at a final concentration of 1% (Metcalfe *et al.*, 1997; Mody *et al.*, 1999; Michelson *et al.*, 2000; Michelson, 2006).

### Quantitative flow cytometric assay

Fixed platelets (50 µL of PRP aliquot, containing approximately 8 × 10^7^µL^−1^ platelets) were incubated undisturbed for 30 min in the dark at room temperature with saturating dilutions (predetermined by titrations) of fluorescein isothiocyanate- (FITC)- and R-phycoerythrin- (R-PE, PE)-labelled monoclonal antibodies. The antibodies used for dual-colour staining were (i) anti-CD41 (mouse anti-human CD41, platelet glycoprotein IIb/IIIa R-PE, Dako Cytomation, Glostrup, Denmark) and anti-A antibody [FITC-conjugated mouse IgG_3_*κ* anti-A monoclonal antibody, clone NaM87-1F6 (Becton-Dickinson Co., BD Pharmingen, San Diego, CA, USA)] and (ii) anti-CD41 and anti-H lectin (lectin, FITC labelled, from *U. europaeus* UEA I, Sigma Chemical Co., St Louis, MO, USA). The quantity of antibody used was sufficient to saturate available A antigen sites: some assays when repeated by incubating the conjugate supernatant with a new platelet aliquot yielded histograms similar to the initial ones.

In order to control the *in vitro* activation, fixed platelets were incubated with anti-CD62p (PE-conjugated mouse anti-human CD62p monoclonal antibody, BD Pharmingen). Any possible neutrophil contamination was discarded by incubation with anti-CD45 (R-PE monoclonal mouse anti-human CD45, leucocyte common antigen, Dako). To detect nonspecific antibody binding, respective isotype controls (negative controls: R-PE-conjugated IgG_1_ mouse monoclonal antibody, Dako; PE-conjugated IgG_1_ mouse monoclonal antibody, BD Pharmingen; and FITC-conjugated mouse IgG_3_*κ*, BD Pharmingen) were also used under the same incubation conditions described earlier.

After staining, platelets were washed (500 ×*g*, 22 °C, 5 min) twice in 0·1 M phosphate-buffered saline (PBS), 0·02% NaN_3_ (w/v), 0·1% bovine serum albumin (BSA) (w/v), pH 7·2–7·4, and resuspended in 500 µL of wash solution (Metcalfe *et al.*, 1997; Mody *et al.*, 1999; Michelson *et al.*, 2000; Michelson, 2006). Platelet-bound fluorescence and control samples (50 µL aliquot of fixed platelets from the same individual) were analysed within 2 h by flow cytometry (FACSCalibur, BD Pharmingen). Forward scatter vs side scatter and forward scatter vs fluorescence gates (FL) of platelets were set. A total of 30 000 events per sample were gated and analysed with the computer software recommended by the manufacturer (CellQuest™ Software, BD Pharmingen).

Results were recorded as the mean fluorescence intensity (MFI) of the platelets positive for both CD41 and either anti-A or anti-H reagents. Maximal platelet activation was considered in the order of ≤1·5% (CD62p expression).

One-colour fluorescence flow cytometry was performed on the RBCs of some individuals. One part of washed RBCs was diluted in two parts of HEPES/BSA-buffered saline and stabilised by fixation in paraformaldehyde at a final concentration of 1% (Metcalfe *et al.*, 1997; Mody *et al.*, 1999; Michelson *et al.*, 2000; Michelson, 2006). An aliquot (5 µL) of fixed RBCs was stained with either anti-A antibody or anti-H lectin. After staining, RBCs were washed twice and resuspended in wash solution before flow cytometry analysis. The results were reported as the MFI in relation to each fluorochrome.

### Serum enzyme activity assays

The 3-*α*-*N*-acetylgalactosaminyltransferase (blood group A GTA) activity was measured in three A_1_ blood group HE family members following the method of Yabe *et al.* (1989), using serum from five A_1_ blood group LE individuals for comparison. In order to assay the GTA activity, 200 µL of 1·6 mm UDP-*N*-acetylgalactosamine (Sigma), 400 µL of 0·1 M cacodylic acid, pH 6·0 (SPI Supplies, West Chester, PA, USA), containing 0·1 M MnCl_2_ (Sigma), and 100 µL of 50% suspension of group O RBCs treated with 0·1% papain solution (Sigma, lot: 127F8075) were added to 1 mL of each serum. The mixture was agitated at 37 °C for 1 h. After incubation, the cells were washed three times in a solution of 0·1 M PBS and 0·5% BSA. A fraction of the converted RBCs was titrated against human anti-A serum. The remainder, after paraformaldehyde fixation, as described earlier, was incubated with FITC-anti-A antibody and tested by flow cytometry.

### Saliva secretion assays

Saliva samples from family members collected in sterile tubes were immediately inactivated by immersion in boiling water for 15 min. After centrifugation (2400 ×*g*, 10 min), quantitative saliva inhibition tests were performed, as published elsewhere (Mallory, 1993).

### Molecular analysis of the *ABO* and *FUT* genes

Some members of the AFB family were subjected to molecular analysis. An initial *ABO* genotyping screen was performed using two independent methods: polymerase chain reaction-restriction fragment length polymorphism (PCR-RFLP) and PCR-allele-specific primer (ASP) (Olsson & Chester, 1995, 1996; Olsson *et al.*, 1998, 2001). Direct DNA sequencing of exons 1–7, as previously described (Olsson *et al.*, 2001), was performed in two samples from family members. In addition, enhancer repeat PCR was used to determine the allele-dependent number of CCAAT-binding factors (CBF/NF-Y) motifs found in this minisatellite region (approximately 4 kbp upstream of the ABO start codon in exon 1) (Irshaid *et al.*, 1999).

The coding regions of the *FUT1* and *FUT2* genes were amplified as one fragment per locus (1171 and 1188 bp, respectively) using the following primers: FUT1·1F, ctcagcctcagagcatttgc; FUT1·3R, gctacttcagaaagtctccctg; FUT2-43F, ccatctcccagctaacgtgtcc; FUT2+43R, gggaggcagagaaggagaaaagg (Yip *et al.*, 2002; Storry *et al.*, 2004). For sequence analysis, the amplified DNA bands were eluted from the gel using the QIAquick gel purification kit (Qiagen Nordic, Crawley, UK) and sequenced directly using an ABI 3130 sequencer (Applied Biosystems, Inc., Foster City, CA, USA) and BigDye^®^ reagents (Applied Biosystems).

### Statistical analysis

Log-transformed (natural logarithms, ln) continuous variables were used, where appropriate. Furthermore, when necessary, the log values were converted to MFI values. The ordinates of the graphics express the expected values for *M* = e^(μ+VAR*/*2)^, and SEM = [(√(e^VAR^− 1)(e^2μ+VAR^)]*/*√(*n*− 1), which are based on the ln (µ and VAR) of the original values read by flow cytometry, where M is the mean MFI value, VAR is the variance and SEM is the standard error of the mean. Groups were compared by Kruskal–Wallis and Mann–Whitney *U* tests. Association between variables was assessed by Spearman's correlation coefficient. Linear regression analysis was used to assess the strength and independence of associations between variables. Categorical variables were compared by *χ*^2^ and Fisher's exact tests. Normality of the distributions was checked by Kolmogorov–Smirnov test. Stability of the MFI values was checked by one-way anova for three correlated samples. This analysis involves each subject being measured in each of the *k* conditions (repeated measures or within-subjects design). A single normal distribution vs two normal mixture or three normal mixture distributions were checked by expectation-maximization (EM) algorithm and by Akaike's information criteria (AIC) (Pawitan, 2001; Burnham & Anderson, 2002). Statistical analysis was performed using spss (version 10·0 for Windows), Analyse-it for Microsoft Excel (version 2·07) and VassarStats software. Differences were considered significant with *P* values <0·05.

## RESULTS

The mean and SD of the population of individual values of MFI were used to define the two empirical categories of A antigen expression on platelets among A_1_ blood group donors: LE, values lower or equal to the mean + 1·96 SD; HE, values higher than the mean + 1·96 SD.

Samples from 241 blood group A donors (A_1_ = 183, A_int_ = 17 and A_2_ = 41) and 50 blood group O donors were obtained. The ln mean + 1·96 SD = 4·82 was used to classify the A_1_ phenotypes: LE = 3·86 ± 0·46 and HE = 5·02 ± 0·17. The prevalence of platelet HE phenotype was estimated in 2% of the A_1_ blood donors.

### Ethnic, age and sex distribution

**Fig. 1:**
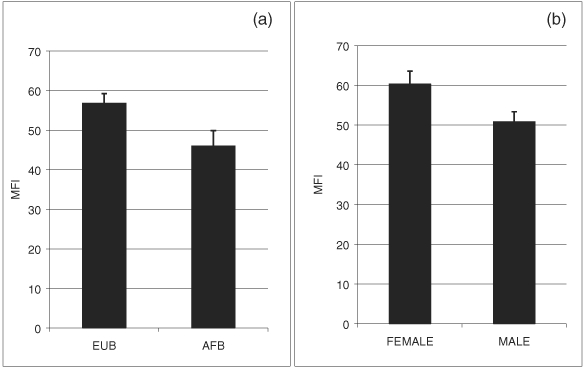
Ethnic and sexual differences. (a) Ethnic differences. The amount (MFI) of A antigen on platelets of the A_1_ subgroup between EUB (*n* = 143) and AFB (*n* = 40) was significantly different (Mann–Whitney = 2111·5; *Z* = 2·53, *P* = 0·0115). No significant differences were found for the A_2_ subgroup (EUB, *n* = 33; AFB, *n* = 8) (Mann–Whitney = 108·0; *Z* = 0·79, *P* = 0·4296). A_int_ subgroup was not analysed due to the small sample size (*n* = 17). (b) Sexual differences. The amount (MFI) of A antigen expressed on platelets of the A_1_ subgroup between male (*n* = 117) and female (*n* = 66) was significant (Mann–Whitney = 2867·5; *Z* = 2·89, *P* = 0·0039). Bars denote ±1 SEM.

Differences in the amount of A antigen expressed on platelets of the A_1_ donors between ethnic and sex subsamples of the population were found to be significant (Fig. 1). There was no correlation between MFI and age for the A_1_ individuals (*P* = 0·5947) (data not shown). Furthermore, the prevalence of HE phenotype distribution between EUB and AFB among A_1_ blood donors was not significant (EUB, LE = 141, HE = 2; AFB, LE = 39, HE = 1; Fisher's exact test, *P* = 0·5251).

### Histogram patterns and comparison of antigen quantity on platelets by expression level

#### A antigen.

**Fig. 2:**
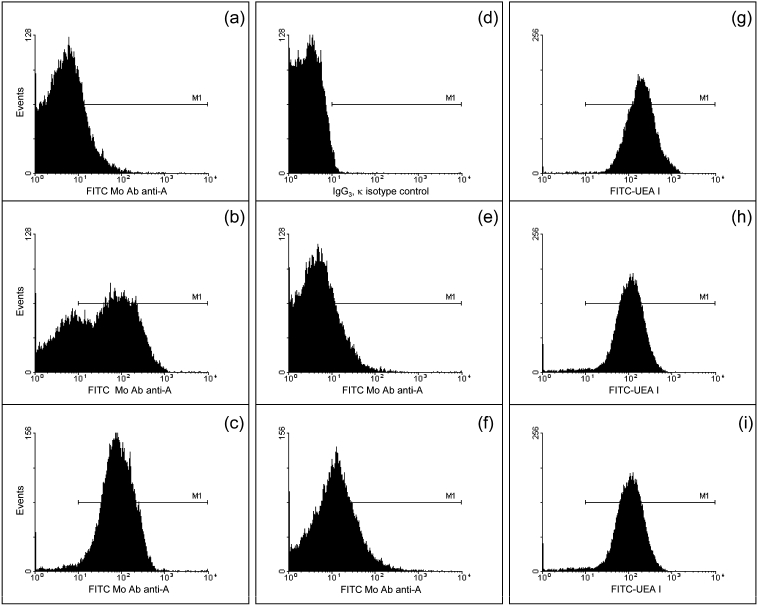
Histogram patterns for the blood group A and H antigen expression on platelet membranes. Platelets were dual labelled with (i) a platelet-specific monoclonal antibody (PE-CD41) and FITC-labelled anti-human blood group A monoclonal antibody (a–c, e and f) and (ii) PE-CD41 and FITC anti-H *U. europaeus* lectin (FITC-UEA I) (g–i). (a and b) The variability patterns of the LE phenotype and (c) the HE phenotype for the A_1_ subgroup. (d) IgG3*κ* isotype control for anti-human blood group A. (e and f) Sample histograms of platelets of A_int_ population. The last column (g–i) shows the same platelet samples shown in a–c exposed to the FITC-UEA I.

Most of the LE platelet phenotype of A_1_ subgroup (62·2%) showed very low MFI and peak channel (PC) profiles, chiefly in the negative region of the distribution, expressing an asymmetric distribution skewed to the right. However, the platelets (PLTs) of almost 30% of the donors showed a wide variation of MFI and PC profiles, ranging from low values near the negative region of the distribution to those lying borderline with the HE platelet phenotype values, denoting a bimodal histogram with high density of A antigen sites. The HE phenotype among A_1_ donors has shown a distinctive separate and symmetric distribution with high MFI and PC profiles, expressing a relatively high density of A antigen sites (Fig. 2a–c).

Most of the platelets of the A_int_ individuals (*n* = 11) have shown profiles very similar to those observed among most of the LE platelet phenotypes of A_1_ subgroup. However, there were a few individuals (*n* = 6) with a slightly higher density of A antigen sites (Fig. 2e,f). The platelets of group O and subgroup A_2_ donors could not be distinguished by visual inspection of the histogram (data not shown).

#### H antigen.

The histogram pattern for all the A variants were similar to a unimodal distribution, reflecting similar and very high MFI and PC profiles (Fig. 2g–i). Both patterns (A and H antigens) were reproducible in blood samples drawn along the time.

### Distribution of the A fluorescence intensity on platelets among subgroups

**Fig. 3:**
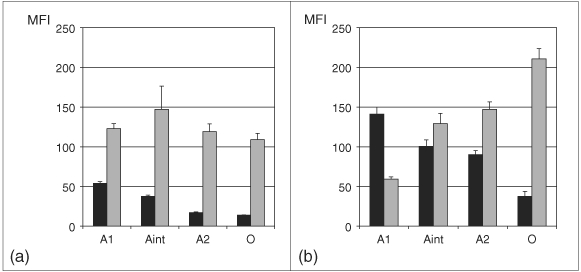
Blood group A and H expression on platelets and RBCs. (a) On platelet surface. The fluorescence intensity on platelets differs significantly among subgroup A_1_, A_2_ and A_int_ donors (Kruskal–Wallis = 90·77, d.f. = 2, *P* < 0·0001) when stained with anti-human blood group A monoclonal antibody (black columns). A decreasing trend of A strength may be observed. The reactivity of *U. europaeus* lectin with platelets (grey columns) does not follow a regular pattern and no significant differences were observed when the H content of the A variants was compared with O blood group donors (Kruskal–Wallis = 1·73, d.f. = 3, *P* = 0·6299). (b) On RBC surface. The fluorescence intensity on RBCs differs significantly among subgroup A_1_, A_2_ and A_int_ donors (Kruskal–Wallis = 50·86, d.f. = 2, *P* = 0·0001) when stained with anti-human blood group A monoclonal antibody (black columns). A decreasing trend of A strength and an increasing trend of H (*U. europaeus*) (grey columns) may be observed. Significant differences were observed when the H content of the A variants was compared (Kruskal–Wallis = 44·69, d.f. = 2, *P* < 0·0001). Bars denote ±1 SEM.

A_1_, A_2_ and A_int_ donors differ significantly among themselves (Fig. 3a). Differences between the MFI on platelets of A_1_ and A_2_ (*P* < 0·0001), A_1_ and A_int_ (*P* = 0·0011), and A_2_ and A_int_ (*P* = 0·0055) donors were also significant. Furthermore, PLTs of A_2_ donors differ significantly from those of O blood group individuals (Mann–Whitney A_2_ vs O 357·0; *Z* = 5·33, *P* < 0·0001). The strength of this reactivity on RBC's control cells showed the same pattern as on platelets, however, with higher values (Fig. 3b).

### Distribution of the H fluorescence intensity on platelets among subgroups

Several control tests were performed with RBCs to verify the standardisation of *U. europaeus* anti-H lectin. These studies confirmed the expected results with the working dilution of the anti-H lectin. The gradient of the H reactivity of RBCs shows the classical results expected for *U. europaeus* lectin (Fig. 3b). However, the H reactivity of the platelets does not follow a regular pattern and no significant differences were observed (Fig. 3a).

### Distribution of A and H MFI within subgroups

**Fig. 4:**
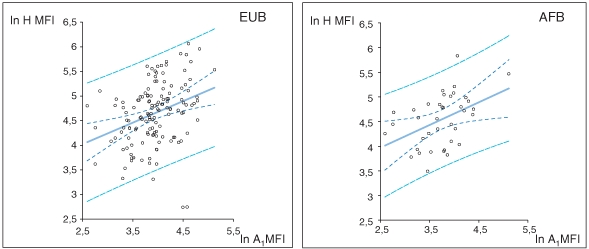
Linear regression of blood group A and H antigen expression on platelets of the A_1_ subgroup. The amount of A and H antigens expressed on platelets of the A_1_ subgroup are positively correlated, i.e. when A antigen increases, H antigen also increases (*P* < 0·001). Shown on the left is the A_1_ blood group of the EUB population. Solid linear fit = 2·934 + 0·436*x*. Shown on the right is the A_1_ blood group of the AFB population. Solid linear fit = 2·823 + 0·4596*x*. Dotted lines represent 95% CI; dashed lines represent 95% prediction interval. The other subgroups did not show correlation between A and H antigens expressed on platelets (A_int_, *P* = 0·1407; A_2_, *P* = 0·6168).

There are two ways to analyse the relationship of A and H content of platelets, one of them was described above comparing the respective amount of intensity of fluorescence among subgroups (Fig. 3a,b). The other approach is to examine the correlation and linear regression of both the parameters. Under this approach, linear regression for both, EUB and AFB, is shown in Fig. 4.

### Variability of MFI individual values

The expresser character may manifest the inherited tendency to remain the same (phenotypic stability) and the tendency to change in response to current environmental conditions. The test for correlated samples is useful in removing the effects of pre-existing individual differences (named SS_wg_ or subjects, extraneous to the principal question of the test), leaving only the error (random variability). The variability inside the samples reflects the fact that there is substantial difference among people with respect to the variable fluorescence intensity. Samples of platelets from 20 individuals of A_1_ subgroup were studied at 0, 30 and 45 days. The results showed that between groups SS = 0·2749, d.f. = 2, MS = 0·1375, *F* = 2·1756, *P* = 0·127479, where SS is the squared sum and MS is the mean square. The variability within days was great; however, when the pre-existing individual differences within each day were removed, the remaining value, the error, which represents the effect of the random variability, and the value of *F* = MS_between_*/*error = 0·1375*/*0·0632 = 2·1756 were not significant. Days 0, 30 and 45 have shown a Gaussian distribution of the ln of the MFI individual values (data not shown). One-way anova for three correlated samples also showed no significant variation (*P* = 0·127479). This result indicated that there was stability of the MFI values, suggesting an inherited condition.

### EM algorithm

**Fig. 5:**
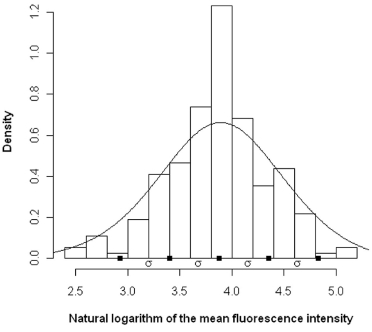
Kernel-smoothed curve of A antigen expression on platelets from A_1_ blood donors. A single normal model, with a mean of the 3·88 ± 0·48 (ln), fitted the data better than a mixture of two or three distributions when submitted to the likelihood ratio test by EM algorithm.

The values for the MFI of A antigen on platelets of each blood group A_1_ individual were subjected to a likelihood ratio test using the EM algorithm (Pawitan, 2001). The results of the likelihood ratio *χ*^2^ test statistics when testing a single normal vs a two-component normal mixture [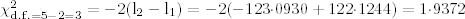, *P* = 0·5 − 0·6] or a three-component normal mixture [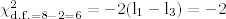 (−123·0930 + 118·6598) = 8·7944; *P* = 0·1 − 0·2] were non-significant and also when comparing a two-component normal mixture and a three-component normal mixture [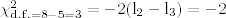 (−122·1244 + 118·6958) = 6·8572, *P* = 0·05 − 0·1], the result was non-significant. The graphic representation is shown in Fig. 5. It was estimated that on average, 98·4% of the ln MFI values, representing the LE platelet phenotype, were lower than or equal to the mean + 2SD, which is in agreement with the normal distribution theory.

### Akaike's information criteria

When comparing a series of models specified *a priori*, the one with the lowest AIC is the ‘best’ one for the data at hand, being a statistical procedure that provides a measure of the goodness-of-fit of a model to a set of data (Burnham & Anderson, 2002). AIC = −2(LL −*p*), where LL is the log likelihood at the maximum likelihood fit and *p* is the number of parameters of the model. It is noteworthy that the same data set was used for each model, i.e. the same observations were used for each analysis. For A_1_ blood donors, the data for a single normal [AIC = −2(−123·0930 − 2) = 250·186] vs a two-component normal mixture [AIC = −2(−122·1244 − 5) = 254·2488] and vs a three-component normal mixture [AIC = −2(−118·6958 − 8) = 253·3916] have also shown that the ‘best’ model was that of one single normal distribution (Fig. 5). These results were similar to those obtained with the generalised likelihood ratio test.

### AFB family results

**Fig. 6:**
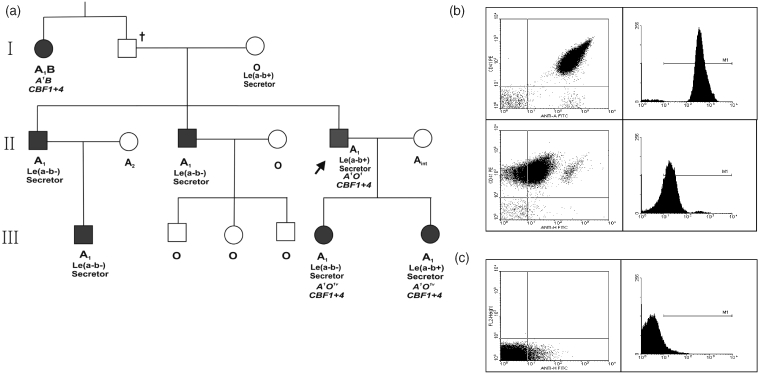
Pedigree of the AFB family and A and H blood group antigen expression in the *propositus* blood sample. (a) Pedigree of the family. The shaded boxes indicate the HE individuals determined by flow cytometry. The phenotypes are presented in normal font, whereas genotypes are presented in italics and the arrow indicates the *propositus*. CBF refers to the upstream enhancer region of the *ABO* gene, and the numbers 1 and 4 reflect the number of sequences within this region, i.e. 1 + 4 would indicate a heterozygote for both a single enhancer unit and a four-repeat enhancer region (see text). (b) Platelets of the *propositus*. Platelets were dual labelled with (i) a platelet-specific monoclonal antibody (PE-CD41) and FITC-labelled anti-human blood group A monoclonal antibody (on the top) and (ii) PE-CD41 and FITC anti-H *U. europaeus* lectin (FITC-UEA I) (on the bottom). (c) RBCs of the *propositus*. RBCs, when exposed to the FITC-UEA I. All HE individuals in this family demonstrated flow cytometry histograms similar to the A_1_ HE *propositus*.

A family study was performed on three generations of relatives of the HE group A_1_*propositus* who demonstrated the largest amount of platelet A antigen. Blood group A antigen levels on the RBCs of these family members were similar to those of five platelet LE AFB donors as measured by serological titrations (titre, *P* = 0·6623; titration score, *P* = 0·8182). However, the serum GTA from the three HE family members tested (II-3, II-5 and III-1) was capable of converting more H antigen on group O RBCs into A antigens compared with the control GTA from A_1_ LE donors, as measured by both serological titrations (log_10_ end-point titre mean = 1·88 vs 1·69; *P* = 0·0145) and flow cytometry (MFI = 33·79 vs 17·93; *P* = 0·0026) (data not shown). All HE family members were secretors, based on either their Lewis phenotypes or their saliva inhibition studies (Fig. 6a).

The comparison between the A and H antigen content of the HE individuals from the population sample with those of the family members has shown that the value of A MFI of the population sample HE (mean = 152·93) falls outside the range of the mean of the HE of the family components [mean = 336·97, 95% confidence interval (CI) 204·80–556·70]. This mean is about 6·4 times higher than the mean of the LE phenotype (52·46). In relation to the H content of the HE expressers, the HE of the family components shows a lower content than the population sample: the mean of the H content of the HE of the family (30·57 ± 5·32) falls outside the 95% CI of the LE fraction of the population (mean = 119·10, 95% CI 109·46–128·44). The expression of the A and H antigens on both, platelets and RBCs, obtained with the *propositus* blood sample, are shown (Fig. 6b,c).

Genomic DNA was available for analysis from 8 of 14 of the living family members including 4 of 7 of the HE family members (I-1, II-5, III-5 and III-6), whose genotypes are indicated in Fig. 6a. By PCR-RFLP and PCR-ASP analyses, all four of these HE members were heterozygous for an *A*^*1*^ allele with its single-unit enhancer region [*A101*, according to the other terminology used in the Blood Group Antigen Mutation Database (http://www.bioc.aecom.yu.edu/bgmut)]. Direct DNA sequencing of all seven exons and the flanking intronic regions of the *ABO* gene in two HE family members revealed a consensus *A*^*1*^*B* (*A101*/*B101*) genotype in one member (I-1) and a consensus *A*^*1*^*O*^*1*^ genotype in the other (II-5); no unexpected polymorphisms were detected. In addition, the entire coding region of both *FUT1* and *FUT2* were also analysed in these two members and no unexpected polymorphisms were identified (Yazer *et al.*, 2005).

## DISCUSSION

In some donors, our studies did not disclose clear-cut differences between the LE and HE-I phenotypes, as described by Curtis *et al.* (2000). The HE-I phenotypes from the point of view of MFI values and also from the histogram patterns cannot always be differentiated from the LE phenotype. For these reasons, we have included both of them as an LE phenotype. On account of these experimental results, we included in the LE platelet phenotype a large spectrum of MFI values.

The A reactivity on platelets follows the same gradient of MFI values as the respective RBC's control cells, which was also described by Cooling (2006).

A positive correlation was observed between A_1_ and H variables (Fig. 4). Our results are similar to those reported by Cooling *et al.* (2005). The extremely low levels of the H content on platelets of the family HE members (in relation to the amount observed in the population sample) (Fig. 6b, on the bottom –*propositus*/ Fig. 2g–i– population sample), together with the almost virtual absence of H antigen on RBCs (Fig. 6c) and the higher levels of serum *α*-GTA, all agree with the HE-II platelet phenotype described by Curtis *et al.* (2000, 2008). Findings made in this family have shown that these platelets carry significantly higher levels of A antigen than other A individuals and have indicated that essentially all H substance, the substrate for synthesis of A antigen, was converted to A by the higher active *α*-GTA.

Furthermore, our results – A_2_ and A_int_ platelet donors have MFI means significantly different from those obtained with platelets from group O individuals – are at odds with other reports which have indicated that the amount of A antigen on most platelets from A_2_ subgroup was very weak or undetectable (Holgersson *et al.*, 1990; Ogasawara *et al.*, 1993; Hou *et al.*, 1996; Curtis *et al.*, 2000; Julmy *et al.*, 2003). An explanation of this difference is apparently not related to ethnic origins. Furthermore, the A strength on platelets suggests that, at least in our population, it may not be appropriate to transfuse platelets from either A_2_ or A_int_ blood donors to group O patients (chiefly, if they receive multiple transfusions for a rather long time), unless these patients have a previous test showing weak or null anti-A agglutinin titration scores.

What remains to be elucidated is the mechanism of inheritance and the factor(s) responsible for producing the HE platelet phenotype. The occurrence of HE in several members suggests familial aggregation. Furthermore, in repeated measures, stability of the MFI values suggesting an inherited condition was observed. Polygenic inheritance – which cannot be excluded as a hypothesis – is demonstrated in the transmission pattern of other continuous or quantitative traits, such as blood pressure (Lander & Schork, 1994). However, the inheritance of the trait was considered to be transmitted as a dominant Mendelian character related to the A or B antigen (Ogasawara *et al.*, 1993; Curtis *et al.*, 2000). We believe that since the first descriptions of the trait (Ogasawara *et al.*, 1993; Curtis *et al.*, 2000; Cooling *et al.*, 2005), the intensity of fluorescence, a typical continuous variable, was described as a practical approximation to a discrete form of two or three phenotypes. The expresser phenotype appears to be cosegregating with the *A*^*1*^ and *B* alleles in the families described by others (Ogasawara *et al.*, 1993; Curtis *et al.*, 2000, 2008) and in this study. However, this cosegregation does not necessarily mean that the *A*^*1*^ and *B* alleles are conditioning the expresser phenotypes. Against the hypothesis of a trait linked to the *ABO* locus, there is the apparent absence of recombinants in the A or B families.

The pattern of inheritance of the HE platelet phenotype demonstrated in the family study (Fig. 6a) suggests autosomal dominant inheritance of the HE phenotype-producing factor(s). Similar pedigrees in Japanese donors demonstrating an inheritance pattern consistent with autosomal dominance have also been reported (Ogasawara *et al.*, 1993). This proposed mode of inheritance whereby the HE-producing factors affect either the GTA or GTB individually explains why, of the 106 AB Japanese donors studied, none demonstrated simultaneously high expression of both A and B antigens on their platelets (Ogasawara *et al.*, 1993), a fact that argues for an *ABO* locus-linked inheritance as opposed to an independent factor able to ‘boost’ the action of any ABO transferase present.

Furthermore, Cooling *et al.* (2005) and the present report found a linear relationship between the amount of A and H antigens on the platelet surface of group A_1_ donors. We hypothesised that a hyperactive 2-*α*-l-fucosyltransferase encoded by the *FUT1* or *FUT2* loci might be responsible for producing the HE phenotype by providing larger quantities of H antigen in secretions and on RBCs, respectively, which could then be converted into group A antigens. However, complete analysis of the *FUT1* and *FUT2* coding regions in two HE individuals revealed only genes without any unexpected polymorphisms consistent with normal H synthesis and thus do not appear to influence the expresser status. All the HE members of the family studied were secretors of A and H substances, thus the passive adsorption of A antigens onto the platelets (Dunstan *et al.*, 1985; Kunicki & George, 1989) cannot be definitively excluded as a contributing factor of the HE state, but it must be realised that based on the frequency of secretor status, this could merely be a coincidence in this family.

The current data set refutes several previously proposed mechanisms of producing the HE phenotype and points to other theories of its inheritance. There are an allele-specific number of minisatellite repeats in the 5′ enhancer region of the *ABO* allele (Kominato *et al.*, 1997; Irshaid *et al.*, 1999). Those enhancers featuring four repeats are associated with higher rates of transcription in luciferase reporter gene experiments (Yu *et al.*, 2000). With this in mind, we decided to analyse the 5′ promoter region in the HE family members for which DNA was available. In an earlier study, which was reported only in abstract form, the presence of the theoretically favourable four-repeat variable number of tandem repeats (VNTR) enhancer region associated with an *A*^*1*^ allele was suggested to cause HE phenotype in two donors (Gershan *et al.*, 2006). To date, this finding has not been presented in full or confirmed by other investigators. Irshaid *et al.* (1999) screened 234 donors of different ethnic/geographic origins and found only a single Jordanian donor who had an *A*^*1*^ allele in *cis* with four repeats. Since then, at least twice as many have been screened and yet no four-repeat enhancers have been found associated with the *A*^*1*^ allele (M. L. Olsson, personal communication). This does not appear to parallel the suggested frequency of HE phenotypes (4–7% in different studies) if the *A*^*1*^ allele with four repeats really had a role in the molecular basis of HE. It should also be noted that there are currently no experimental data to support the notion that enhancer repeats are important for *ABO* transcription in haematopoietic tissue. The studies suggesting that they do in fact play a role in regulating the rate of transcription (and thereby theoretically, e.g. the A or B expression on platelets) were performed in cell lines of gastric cancer origin (Kominato *et al.*, 1997; Yu *et al.*, 2000).

In this study, the expected single-unit enhancer region associated with the *A*^*1*^ allele of HE individuals, the exons 1–7, including splice sites and the putative proximal promoter region had no unexpected mutations/polymorphisms. On the contrary, a closer analysis of the current data reveals some interesting phenomena that might not necessarily be accounted for by a simple inheritance pattern. In spite of the consensus *ABO* gene in the current platelet HE family members (I-1 and II-5 individuals), the serum GTA from other HE family members (II-3 and III-1 individuals) synthesised more A antigens on group O cells compared with the serum GTA from A_1_ LE control donors. The higher than normal GTA activity has also been previously reported in other platelet HE individuals (Ogasawara *et al.*, 1993; Curtis *et al.*, 2000), but it is still unclear if this finding is due to a higher concentration of serum GTA or a qualitatively enhanced enzyme at the same concentration as in LE individuals. Yet another possibility to explain the varying ABO antigen levels on platelets is megakaryocyte-related interindividual differences.

The cause of the platelet phenotype remains elusive, although its aetiology probably lies outside the *ABO* locus itself. We have presented data that support both a single and polygenetic explanation for this phenotype; further research is warranted to more fully elucidate the cause of this fascinating and potentially clinically relevant phenomenon.
